# Germ Cell Tumors in Dysgenetic Gonads

**DOI:** 10.6061/clinics/2019/e408

**Published:** 2019-11-04

**Authors:** Mauri José Piazza, Almir Antonio Urbanetz

**Affiliations:** I Departamento de TocoGinecologia, Universidade Federal do Parana, Curitiba, PR, BR

**Keywords:** Ovarian Neoplasms, Gonadal Dysgenesis, Gonadal Neoplasms, Gonadoblastoma, Germ Cells Tumors, Dysgerminoma, Teratoma, Yolk-sac Tumor

## Abstract

This review describes the germ cell neoplasms that are malignant and most commonly associated with several types of gonadal dysgenesis. The most common neoplasm is gonadoblastoma, while others including dysgerminomas, yolk-sac tumors and teratomas are rare but can occur. The purpose of this review is to evaluate the incidences of these abnormalities and the circumstances surrounding these specific tumors.

According to well-established methods, a PubMed systematic review was performed, to obtain relevant studies published in English and select those with the highest-quality data.

Initially, the first search was performed using gonadal dysgenesis as the search term, resulting in 12,887 PubMed papers, published, from 1945 to 2017. A second search using ovarian germ cell tumors as the search term resulted in 10,473 papers, published from 1960 to 2017. Another search was performed in Medline, using germ cell neoplasia as the search term, and this search resulted in 7,560 papers that were published between 2003 to 2016, with 245 new papers assessing gonadoblastomas.

The higher incidence of germ cell tumors in gonadal dysgenesis is associated with a chromosomal anomaly that leads to the absence of germ cells in these gonads and, consequently, a higher incidence of neoplasms when these tumors are located inside the abdomen. Several hypotheses suggest that increased incidence of germ cell tumors involves all or part of the Y chromosome or different genes.

## INTRODUCTION

Germ cell tumors (GCTs), or germ cell cancers (GCCs) are a heterogeneous group of ovarian neoplasms that originate, during different stages of development, from germ cells that colonize the ovaries or gonads. These specific tumors account for approximately 15-20% of primary ovarian tumors, 95% of which are mature cystic teratomas. They predominantly affect young women and may differ in their clinical, histologically and biologically presentation and include benign and malign subtypes. Nearly 60% of ovarian tumors occurring in women under the age of 21 and less than 5% of ovarian cancers have a germ cell origin (1).

The risk for the development of GCTs is an important factor in the management of patients with disorders of sex development (DSD) (2).

## METHODS

We performed a PubMed systematic review, to obtain relevant studies that were published in English and select those with the highest-quality scientific data.

The first search was performed using gonadal dysgenesis as the search term, resulting in 12,887 papers in PubMed, published from 1945 to 2017. The second search using ovarian GCTs as the search term resulted in 10,473 papers, published from 1960 to 2017.

Other papers describing GCTs were as follows: gonadoblastomas, published between 1953 and 2017 (790 papers); dysgerminomas, published between 1944 and 2017 (2208 papers); teratomas, published between 1913 and 2017 (5573); and Yolk-sac tumors, published between 1945 and 2017 (756 papers).

Another search was performed with Medline using germ cell neoplasia as the search term, resulting in 7560 papers that were published between 2003 and 2016; 245 new papers on gonadoblastomas were published.

Inclusion criteria: Meta-analyses, systematic reviews and studies with a large number of patients or with relevant findings.

Exclusion criteria: Papers that did not meet the above criteria.

## GONADAL DYSGENESIS

Gonadal dysgenesis comprises a vast array of clinical conditions with different somatic and genetic features. Special attention is necessary because in some of these cases, neoplasia can develop in dysgenetic gonads (3).

The different classifications for gonadal dysgenesis proposed by the authors of this review are based on the abnormal differentiation of the gonads, and these classifications include three categories, presented in Table 1.

Table 2, in 2006, the Chicago consensus meeting on the management of the DSD classification was established and revised by Hughes et al. (4).

In genetic females or individuals presenting with X chromosome monosomy, the ovaries do not undergo complete differentiation and these undifferentiated gonads are similar to a white “conjunctive streak”, usually having no endocrine function or gamete production (5).

This newly recognized “undifferentiated gonadal tissue” has been demonstrated to be a gonad with a high risk for the development of a tumor, including gonadoblastoma and other tumor types.

Here, we analyze this condition in different groups and determine the groups that present a greater risk for developing neoplasia in their dysgenetic gonads.

## GONADAL DYSGENESIS AND SOMATIC ABNORMALITIES

Turner syndrome was previously described by Turner (6) as a distinct entity, with clinical manifestations including the “stigmas” that are pathognomonic of this entity and a hormonal analysis indicating hypergonadotropic hypogonadism.

Genetic analysis reveals a karyotype of 45,X in 60% of cases but other chromosomal abnormalities might be detected, including mosaicisms associated with the 45,X cell lineage; 45,X/46,XX cell lineage; or 45,X/47,XXX cell lineage.

On the other hand, karyotype analysis is crucial for determining whether or not the Y chromosome is present, and whether or not the chromosome is complete or structurally abnormal, containing deletions such as 45,X/46,X(Yq) or 45,X/46,Xi(Yp) (7).

Lippe described how germ cells usually migrate to the germinal ridges of the embryo until the third month of gestation. From this point, a degeneration process occurs, resulting in the elimination of germ cells and concurrently accelerating the fibrosis process of the gonadal stroma (5). In 45,X monosomy (Turner syndrome), there is normal ovarian development at the beginning of fetal life followed by a rapid loss of germ cells beginning at the 22^nd^ week of gestation, resulting in a streaked gonad around the time of delivery (8).

This abnormality in gonadal organogenesis is associated with a greater risk for developing neoplasia in the dysgenetic gonads (9).

### Pure Gonadal Dysgenesis

Hormonal analysis also consists of a hypergonadotropic hypogonadism entity with conjunctive-streak gonads and a 46,XX or 46,XY karyotype.

These patients have a female phenotype with normal Mullerian structures but are considered infantile due to their lack of adequate hormonal stimulation.

The 46,XX form of pure gonadal dysgenesis results from a gene mutation, which is classified as an autosomal recessive disorder.

Therefore, Swyer’s description of the 46,XY form of pure gonadal dysgenesis is clinically identical to the previous description. Here, dysgenetic gonads are at a 30% risk of developing a neoplasia, such as gonadoblastoma or dysgerminoma (2).

The potential genetic etiology the Swyer form is due to an autosomal gene disorder, with recessive or dominant features, but is restricted to males (10).

Previous studies by Verp and Simpson et al., Scherer et al. and Assumpção et al. reported that 10-15% of cases of this gonadal dysgenesis are attributed to a mutation or deletion in SRY, but 70-80% of cases are of unknown etiology (9,11,12).

### Mixed Gonadal Dysgenesis

Mixed gonadal dysgenesis a rare disorder characterized primarily by a dysgenetic streaked gonad on one side and a potentially undifferentiated testicle on the other (13).

Therefore, Mullerian structures develop on one side and are inhibited by the presence of anti-Mullerian hormone (AMH) that is produced by the Sertoli cells of the testicle.

The external genitals are subject to variable levels of androgenization, which depend on testicular androgen activity. A wide variety of clinical manifestations can occur, ranging from male external genitalia with varying degrees of hypospadias, to ambiguous or even female genitalia (14).

Mixed gonadal dysgenesis (MGD) is also known as asymmetric or atypical gonadal dysgenesis, and genetic studies have revealed a karyotype of 45,X/46,XY mosaicism.

Turner syndrome “stigmas” can be frequently found among these patients (13). In previous studies, Verp et al., Alvarez-Nava et al., and Mendez et al. reported that dysgenetic gonads or even dysgenetic testicles are at a greater risk for developing neoplasia (9,13,14).

### Denys-Drash Syndrome

Denys-Drash syndrome is a condition that is characterized by a typical male chromosome pattern (46,XY), renal failure and Wilms tumors, whereas the external genitalia range from ambiguous to completely female or completely male (15).Dysgenetic gonads or rudimentary and immature testes can be observed. Therefore, the internal genitals of affected individuals can develop Wolffian or Mullerian structures. The short arm of chromosome 11 from the 11p13 chromosomal region harbors the WT1 tumor suppressor gene, which was previously reported to be the oncogene hypothesized to result in Wilms tumors (16). As a result, there is also a greater risk for developing gonadal tumors, such as gonadoblastoma.

## GONADAL TUMORS

Abnormalities in gonadal development are triggering factors for the development of neoplasia in dysgenetic gonads. A variety of different tumors can develop, and these tumors are frequently classified as germinal tumors, also known as GCCs (17). The malignant germ cell tumor is a complication that can occur in patients with a DSD. The development of tumors in an individual depends on a number of genetic or epigenetic factors related to early gonadal and germ cell development and possibly correlates with genetic susceptibility.

Gonadal GCTs are histologically subdivided into seminomatous and nonseminomatous tumors. The seminomatous tumors are composed of neoplastic cells that are arranged in a similar manner to embryonic germ cells or gonocytes, and these tumors are classified as dysgerminomas in the ovary or dysgenetic gonad, as seminomas in the testis and as germinomas in extragonadal localizations. Nonseminomatous neoplasia comprises different types of tumors including teratomas, yolk-sac tumors, choriocarcinomas and embryonal carcinomas (18,19). The gonadoblastoma is the precursor to the invasive germ cell tumor, predominantly dysgerminoma, in the dysgenetic gonads.

### Gonadoblastoma

Gonadoblastoma is a mixed germ cell tumor with undifferentiated cells and is most frequently observed in female children, 1/3 of whom are younger than 15 years of age. This tumor was first described by Scully (20). It is most commonly found on the right gonad, although it has been frequently detected on both sides (21). This tumor is regarded as an *in situ* type of germ cell tumor in dysgenetic gonads and is a precursor to seminoma/dysgerminoma tumors (22).

Affected patients present with delayed puberty with some virilization as a result of androgen production. Tumors can grow from histological lesions up to a maximum of 8 cm. Most of them measure between 1 and 5 cm, but a considerable proportion are microscopic in size. A suspicious case can be confirmed by the presence of microcalcifications in the gonads (20).

Histologically, this neoplasia consists of a tumor composed of two cell lineages, namely, germ cells that are similar to dysgerminoma cells and sex cord cells that are similar to immature Sertoli cells or granulosa cells. The stromal tumor may also carry cell nests that are similar to Leydig cells and that contain Reinke crystalloids. Such cell “nests” are usually small, solid, and oval or round-shaped, and may have inner calcifications. Because of this mixture of a germ cell tumor and undifferentiated cells, 60% of gonadoblastoma cases can result in or be associated with other types of neoplasms, such as dysgerminomas, although gonadoblastomas are benign tumors that do not metastasize (23,24). Pure gonadoblastomas are regarded as an *in situ* type of germ cell tumor that affects patients with dysgenetic gonads.

Scully reported that the Y chromosome found in the karyotypes of affected patients was the triggering factor for the development of gonadal neoplasia (25).

Specific loci from genes identified at the Y chromosome are considered to be the trigger for the development of tumors. These genes are probably located in the short arm of this chromosome, within its centromere (26).

In 1987, Page postulated that there was a gonadoblastoma locus on the Y chromosome, which was located near the centromere or on its long arm (27). Tsuchiya et al. and Salo et al. detected a small and distinct region within the centromere of the Y chromosome with an estimated size of 1-2 Mb (26,28). The testis-specific protein, Y-encoded (TPSY) gene, which is located within the gonadoblastoma locus on the Y chromosome, has been shown to be involved in the numerous transformation processes of germ cells (28).

The identification of these genetic markers in the Y chromosome is an important predictor of future neoplasm development.

Bagnoli et al. and Piazza et al. have previously described the incidence of neoplasms in dysgenetic gonads, revealing their concern in view of this perspective (3,21). Patients with gonadoblastoma without dysgerminoma or another germ cell tumor are treated only by surgical excision of the gonads without any additional therapy. However, if dysgerminoma or another tumor is present, surgical staging and postoperative combination chemotherapy with bleomycin, ectoposide and cisplatin may be required (29).

The true incidence of gonadal GCTs in children is unknown, and few studies have been published on the incidence of this tumor in pediatric populations. When the correct diagnosis and proper treatment occur during the early stage, most gonadal tumors are curable (30).

Bianco et al. observed that SRY or OCT4 gene expression increased the risk of developing gonadoblastoma, even in patients with Turner syndrome (31-33). De Marqui et al. observed and described the prevalence of the Y chromosome with gonadoblastoma in Turner syndrome, and molecular analysis to determine Y chromosome sequences in Turner syndrome is indicated, regardless of karyotype (34).

### Dysgerminoma

Dysgerminoma is a malignant tumor that is driven by the proliferation of primitive germ cells associated with conjunctive tissue septum. It is the second most common neoplasm associated with gonadoblastoma or that develops in dysgenetic gonads. This tumor is identical to testicular seminoma and is frequently diagnosed in the second decade of life (35).

Macroscopically, these are well-encapsulated and unilateral tumors in 90% of cases. Ten percent of cases have contralateral involvement, and another 10% of cases have occult involvement that can only be detected through biopsies (36).

Histological analysis reveals the proliferation of germ cells with a polygonal or oval shape, which linked to chords with a vacuous, abundant and pale cytoplasm. Chronic T lymphocyte infiltration is also present, and a variable mitotic rate, as well as anisokaryosis rate, define the degree of malignancy (37).

No factors have been associated with the etiology of dysgerminoma, apart from an increased incidence associated with dysgenetic gonads. A total of 5% of dysgerminomas are associated with cytogenetic abnormalities involving all or part of the Y chromosome or MGD, namely, 45,X/46,XY (34,38).

Capito et al. have reported the rare occurrence of this tumor in association with gonadoblastoma in 6 children, who were approximately 11 years of age, presenting with pure gonadal dysgenesis and the 46,XY karyotype (37). Tumoral behavior, regardless of whether or not trophoblastic tissue producing human chorionic gonadotropin (hCG) is present, requires further examination and follow up. Other references that relate dysgerminomas to gonadoblastomas have been reported by Yilmaz et al., Maleki et al., Talerman et al., and Subbiah et al. with descriptions of several anatomopathological, chromosomal and gene anomalies (23,24,35,38). At the time of surgical operation, this neoplasm is found to have spread locally in 20 to 30% of cases. The main route of dissemination is through regional lymphatics, and the most commonly involved lymph nodes are the retroperitoneal, paraaortic and mediastinal lymph nodes. Bony metastases may occur in the sacral, lumbar or dorsal vertebrae (39).

Brody analyzed the factors that affect patient survival and concluded that the five-year survival rate was 95% if the tumor was encapsulated and movable, 75% if adhesions were present, 60% if rupture of the tumor capsule occurred, and only 33% if metastases were detected (40). Most recurrences are found in the first two years after the primary operation, and the recurrence rate depends on the extent of the lesion and the type of therapy (36).

### Endodermal Sinus Tumor

The endodermal sinus tumor is a morphologically heterogeneous tumor and is, also known as a yolk-sac tumor. The endodermal sinus tumor arises from endodermal structures and is characterized by alpha-fetoprotein production and its highly malignant nature. Among other indicators, the malignant nature of this tumor serves as an epithelial cell lining marker for these tumors (41).

These tumors are relatively well-encapsulated tumors, that are gray in color, have a soft consistency, and have central necrotic areas, and the largest diameters of these tumors vary from 8-25 cm (42).

Histological analysis shows reticular features with arborescent-like stromal tissue that includes several degrees of atypia, with Schiller-Duval bodies considered pathognomonic of this neoplasm (43).

Positive cytokeratin tests can differentiate solid yolk-sac tumors from dysgerminomas (43).

This neoplasm may manifest in childhood when it has a soft consistency and is more sensitive to chemotherapy. During adulthood, tumors have a mixed appearance and a less favorable chemotherapeutic response (30). These tumors can be found either on dysgenetic gonads or on other body parts, such as the testes, vagina, ovaries, urachus, retroperitoneum, or mediastinum. Cytogenetic analysis revealed the increased incidence of this neoplasm in isochromosome i(12p) or in 1p36 deletions (44). Likewise, it was observed that the loss of RUNX3 gene located at chromosome 1p36.1 triggered tumor development, since this gene serves as a tumor suppressing agent (45).

Ito & Kawamata, Madiwale et al., Nam et al., Riopel et al. and Looijenga et al. previously described this neoplasm in patients with gonadal dysgenesis (44-48). Nasioudis et al. analyzed 561 women with this neoplasia and determined that the median age was 23 years and that the majority of tumors, namely, 58.5%, were stage I or stage II disease, while 29.6% and 11% of tumors were stage III and stage IV disease, respectively. These patients were treated by hysterectomy, lymphadenectomy and omentectomy, and those who received chemotherapy achieved an increased survival rate compared with the group that did not receive chemotherapy (30).

Other neoplasms, including teratomas and juvenile granulosa cell tumors, have also been described as potential gonadal dysgenesis tumors (48).

Teratomas, commonly known as mature cystic teratomas, are cystic tumors and consist of a monodermal teratoma component. Generally arising from the epithelium, these tumors are frequently referred to as “dermoid cysts” and occur during the first years of menacme, representing approximately 58% of benign ovarian tumors. Ovarian teratomas are bilateral in 10-17% of cases, always contain brownish-gray and always contain greasy material and hairs (49).

Neoplasms that contain other benign tissues can also be detected, and these neoplasms include teratomas composed of thyroid tissue, namely, “struma ovarii”; carcinoid tumors; and immature teratomas known as adenomas that produce corticotrophin or prolactin (50).

These neoplasms represent 3% of all cases and 1% of all ovarian carcinomas and are often unilateral and solid (50).

Its markers include the beta subunit of hCG, alpha-fetoprotein (AFP) and plasma T4 (thyroxine). Norris et al. classified immature teratomas into grades 1, 2 and 3 according to their cell differentiation status (50).

Immature grade 1 or 2 teratomas are diploid in 90% of cases; immature grade 3 teratomas are aneuploid in 66% of cases or present other karyotype abnormalities (50).

Embryonal carcinomas or polyembryomas are rare tumors that can develop simultaneously from the germ cell component of gonadoblastomas. Given the wide variety of hormones produced, its clinical manifestations are numerous and may include precocious pseudopuberty during childhood or abnormal uterine bleeding during menacme (51).

According to Troche & Hernandez, the incidence of such neoplasms is 9.8% (52).

## RISKS FACTORS for GERM CELL CANCERS (GCCs) in DSD PATIENTS

The risk for gonadal GCC development in DSD depends on a large number of factors that are involved in damage to the gonads.

The gonadoblastoma-Y (GBY) region is a prerequisite for gonadoblastoma and is located on the Y chromosome. A study by Cools et al. hypothesized that a testis-specific protein encoded by the GBY gene on the Y chromosome may have an oncogenic function at high levels (26,28,53,54). Patients with Turner syndrome have an increased risk of GCC due to the hidden presence (mosaic) of the GBY region; additionally, patients with Klinefelter syndrome exhibit early apoptosis of germ cells, and in Klinefelter syndrome, only the mediastinum and brain have a high risk for developing an aggressive GCC (55,57).

This increased risk is determined by the presence of Y material, mosaicism of the genotype and the degree of masculinization.

Newer molecular methods permit the detection of Y chromosome material. FISH and X and Y centromere probes are sometimes recommended to determine the origin of ring or small marker chromosomes. Currently, real-time polymerase chain reaction with multiple Y specific probes is more sensitive than the FISH technique and can detect Y mosaicism in up to 12% of Turner syndrome cases (58).

The rate of gonadoblastoma for Turner syndrome with the Y chromosome that was detected by PCR or FISH varied from 4-60% in 14 studies, and when the data are compiled, the total rate is 10% (59).

Gravholt et al. in 2000 study described a Gonadoblastoma occurrence in female with Turner syndrome and Y chromosome material. In 114 females with phenotype Turner syndrome were detected 14 (12.2%) who had Y chromosome, although seven of these patients had a karyotype that did not suggest the presence of Y chromosome material. In 10 of these patients the presence of Y material were detected by PCR with many primers: SRY, ZFY, DYZ, DYS132 and DYZ1. The risk to develop gonadoblastoma has been estimated to be larger than 30% (60).

Regarding the localization of the gonads, there are related small series about the testis. There is a lower incidence of GCC when the testes are in a normal scrotal position. Klaassen et al., demonstrated that GCC with a seminomatous histology is more common in undescended testes than in scrotal testes (61).

Regarding androgen, the absence of androgen receptor and the presence of complete androgen insensitivity syndrome protect against the risk for developing GCC because of the resulting lack of androgen signaling that leads to the apoptosis of germ cells and inhibits spermatogenesis, as concluded by Cools et al. and by O’Shaughnessy et al. (54,62).

Regarding the genes involved, numerous genes and transcription factors influence the development of the urogenital ridge and the testis. Germ cells originate in the yolk-sac, and migration occurs to develop the urogenital ridge and the gonads.

OCT4 (octamer-binding-protein), also known as OCT3 or POU5F1 is a transcription factor with a fundamental function in the pluripotency of embryonic stem cells and primordial germ cells. It has been proposed as a marker for GCTs with pluripotency and is specific to seminoma/dysgerminoma and embryonal carcinoma (63). Palma et al. analyzed the utility of OCT3/4, TSPY and beta-catenin as biological markers for gonadoblastoma formation and malignant GCTs, and previous results suggested that these markers participate in important steps during GB malignant transformation (64). With the development of this method, it became capable of revealing the increasing level of OCT4 expression in a wide variety of gonadal and extragonadal GCTs.

The formation of early undifferentiated gonads is regulated by a number of genes, including steroidogenic factor 1 (SF1), Wilms tumor 1 (WT1), and chromobox homolog 2 (CBX2) (65). Ovarian development was thought to be a passive process that was driven only by the absence of SRY expression; however, it was recently observed that ovarian development requires the involvement of WNT family member 4 (WNT4), Forkhead Box L2 (FOXL2), beta-catenin 1 (CTNNB1), and R-spondin1 (RSPO1) (61). Other markers have been identified, including SOX17 in seminomas and SOX2 in embryonal carcinomas (66).

Specific WT1 mutations are capable of causing Denys-Drash syndrome and Frasier syndrome, with an increased risk for Wilms kidney tumor and gonadal dysgenesis (15,16).

The Genome wide association studies (GWASs) were used to identify multiple factors associated with familial testicular GCC risk including a large number of high risk single nucleotide polymorphisms whose levels were increased; the promoter methylation of several genes namely, PDE11A, SPRY4 and BAK1; and decreased promoter methylation of KITLG (Stem Cell Factor-c-kit System) (67-69).

Gonadal examination and biopsy must be performed multiple times to analyze the tissue during gonadectomy or to postpone the gonadectomy. This is important to balance the potential risk for GCC or to endocrine and fertility function.

Histological and immunohistochemical analyses need to be performed by a pathologist who understands gonadal development and DSD (70).

## CONCLUSIONS

We conclude that the higher incidence of GCTs in gonadal dysgenesis results from the following.

1. Chromosomal gene anomalies that lead to the absence of germ cells in these gonads and, consequently, a higher incidence of neoplasms when located inside the abdomen. The location of this undifferentiated gonadal tissue at a site with an abnormal temperature can result in metabolic rate changes and, therefore, an increased risk of developing neoplasia. Our hypothesis is that mutated genes can alter the antigen related cell surface in embryonic cells, or result from a lineage of aneuploid cells arising from germ cells and that the mutated genes can be tumor suppressor genes (54).
  This undifferentiated gonadal tissue that is found in dysgenetic gonads would be naturally more susceptible to the development of neoplasia (70).
2. Together with the centromere and the region near the long arm of the Y chromosome, a DNA region of 1-2 Mb coinciding with the GBY region acts as a determinant gene for tumor growth (26).
3. The prevalence of GCTs is increased in patients with DSD containing Y chromosome material in their karyotype and due to the presence the TSPY gene (71).
4. Gonadal biopsy and immunohistochemical biomarkers (OCT3/4 (POU5F1), TSPY, SOX9, FOXL2 and KITLG (SCF)) are important to characterize situations in which GCC development is possible (germ cells tumors) (72).
5. One specific marker for gonadoblastoma and germ cell neoplasia *in situ* (GCNIS) is POU5F1, and prolonged expression of this marker is crucial to GCC development (73).
6. When all or part of the Y chromosome is detected in these patients, bilateral gonadectomy must be performed. An increased prevalence of GCTs has been noted in individuals with a Y sequence who have Turner syndrome.

## AUTHOR CONTRIBUTIONS

All authors participated in the review of the literature and in the writing of the manuscript.

## Figures and Tables

**Table 1:** Proposed criteria for the classification of gonadal dysgenesis.

1. Male Phenotype	Klinefelter Syndrome. 47,XXY or Similar
2. Female Phenotype	a - Gonadal Dysgenesis with Somatic Abnormalities: 45,X or Similar
	b - Pure Gonadal Dysgenesis: 46,XX or 46,XY (Swyer Syndrome)
3. Female/Ambiguous/Male Phenotype	Mixed Gonadal Dysgenesis: 46,XY/45,X Denys-Drash Syndrome: 46,XY

**Table 2:** Classification of disorders of sex development (modified from Hughes et al. 2006) (4).

Sex chromosomal DSD	46,XY DSD	46,XX DSD
45,X Turner syndrome	Testicular GD	Ovarian GD
47,XXY Klinefelter syndrome	Complete GD	Ovotesticular DSD
45,X/46,XY and variants	Partial GD	Testicular DSD
46,XX/46,XY and variants	Gonadal regression	Androgen excess
	Ovotesticular DSD	Fetal level CAH
	Disorder of androgen	Aromatase deficit
	Biosynthesis/action	Maternal ovary tumor
	LH receptor defect	Fetal adrenal tumor
	Defective action AMH	Other: cloacal extrophy
	or AMH receptor	Severe epispadias
	Other: cloacal exstrophy	Mayer-Rokitansky-Kuster-Hauser syndrome
	Severe hypospadias	

AMH=Anti-Mullerian hormone CAH=Congenital adrenal hyperplasia

DSD=Disorder of sex development GD=Gonadal dysgenesis

## References

[1] Koonings PP, Campbell K, Mishell DR, Grimes DA (1989). Relative frequency of primary ovarian neoplasms: a 10-year review. Obstet Gynecol.

[2] Gourlay WA, Johnson HW, Pantzar JT, McGillivray B, Crawford R, Nielsen WR (1994). Gonadal tumors in disorders of sexual differentiation. Urology.

[3] Bagnoli VR (1984). Análise do potencial oncogênico da gonada disgenética na disgenesia gonadal. Ginecol Obstet Bras.

[4] Hughes IA, Houk C, Ahmed SF, Lee PA, LWPES Consensus Group; ESPE Consensus Group (2006). Consensus statement on management of intersex disorders. Arch Dis Child.

[5] Lippe B, Sperling MA, editor (1996). Turner syndrome, In. Pediatric endocrinolology.

[6] Turner HH (1938). A syndrome of infantilism, congenital webbed neck, and cubitus valgus. Endocrinology.

[7] Oliveira RM, Verreschi IT, Lipay MV, Eça LP, Guedes AD, Bianco B (2009). Y chromosome in Turner syndrome: review of the literature. Sao Paulo Med J.

[8] Modi DN, Sane S, Bhartiya D (2003). Accelerated germ cell apoptosis in sex chromosome aneuploid fetal human gonads. Mol Hum Reprod.

[9] Verp MS, Simpson JL (1987). Abnormal sexual differentiation and neoplasia. Cancer Genet Cytogenet.

[10] De Mello MP, Assumpção JG, Hackel C (2005). Genes envolvidos na determinação diferenciação do sexo. Arq Bras Endocrinol Metab.

[11] Scherer G, Held M, Erdel M, Meschede D, Horst J, Lesniewicz R (1998). Three novel SRY mutations in XY gonadal dysgenesis and the enigma of XY gonadal dysgenesis cases without SRY mutations. Cytogenet Cell Genet.

[12] Assumpção JG, Benedetti CE, Maciel-Guerra AT, Guerra G, Baptista MT, Scolfaro MR (2002). Novel mutations affecting SRY DNA-binding activity: the HMG box N65H associated with 46,XY pure gonadal dysgenesis and the familial non-HMG box R301 associated with variable phenotypes. J Mol Med.

[13] Alvarez-Nava F, Gonzalez S, Soto S, Pineda L, Morales-Machin A (1999). Mixed gonadal dysgenesis: a syndrome of broad clinical, cytogenetic and histopathologic spectrum. Genet Couns.

[14] Méndez JP, Ulloa-Aguirre A, Kofman-Alfaro S, Mutchinick O, Fernández-del-Castillo C, Reyes E (1993). Mixed gonadal dysgenesis: clinical, cytogenetic, endocrinological, and histopathogical findings in 16 patients. Am J Med Genet.

[15] Mueller RF (1994). The Denys-Drash syndrome. J Med Genet.

[16] Hastle ND (1992). Dominant negative mutations in the Wilms tumour (WT1) gene cause Denys-Drash syndrome-proof that a tumour-suppressor gene plays a crucial role in normal genitourinary development. Hum Mol Genet.

[17] Abell MR, Johnson VJ, Holtz F (1965). Ovarian neoplasms in childhood and adolescence. I. Tumors of germ cell origin. Am J Obstet Gynecol.

[18] Oosterhuis JW, Looijenga LH (2005). Testicular germ-cell tumours in a broader perspective. Nat Rev Cancer.

[19] Oosterhuis JW, Stoop H, Dohle G, Boellaard W, van Casteren N, Wolffenbuttel K (2011). A pathologist’s view on the testis biopsy. Int J Androl.

[20] Scully RE (1953). Gonadoblastoma; a gonadal tumor related to the dysgerminoma (seminoma) and capable of sex-hormone production. Cancer.

[21] Piazza MJ, Teixeira AC, Amaral MF, Netto AS, Marçallo FA (1977). Bilateral gonadoblastoma with chromosomal aberration. Obstet Gynecol.

[22] Cools M, van Aerde K, Kersemaekers AM, Boter M, Drop SL, Wolffenbuttel KP (2005). Morphological and immunohistochemical diferences between gonadal maturation delay and early germ cell neoplasia in patients with undervirilization syndromes. J Clin Endocrinol Metab.

[23] Yilmaz B, Gungor T, Bayramoglu H, Soysal S, Mollamahmutoglu L (2010). Bilateral ovarian gonadoblastoma with coexisting dysgerminoma in a girl with 46, XX karyotype. J Obstet Gynaecol Res.

[24] Maleki Z, Loveless M, Fraig M (2011). Coexistence of gonadoblastoma and dysgerminoma in a dysgenetic gonad on touch preparation: a case report. Diagn Cytopathol.

[25] Scully RE (1981). Neoplasia associated with anomalous sexual development and abnormal sex chromosomes. Pediatr Adol Endocrinol.

[26] Tsuchiya K, Reijo R, Page DC, Disteche CM (1995). Gonadoblastoma: molecular definition of the susceptibility region on the Y chromosome. Am J Hum Genet.

[27] Page DC (1987). Hypothesis: a Y-chromosomal gene causes gonadoblastoma in dysgenetic gonads. Development.

[28] Salo P, Kaariainen H, Petrovic V, Peltomaki P, Page DC, de la Chapelle A (1995). Molecular mapping of the putative gonadoblastoma locus on the Y chromosome. Genes Chromosomes Cancer.

[29] Nasioudis D, Chapman-Davis E, Frey MK, Caputo TA, Holcomb K (2017). Management and prognosis of ovarian yolk sac tumors: an analysis of the National Cancer Data Base. Gynecol Oncol.

[30] Lin X, Wu D, Zheng N, Xia Q, Han Y (2017). Gonadal germ cell tumors in children: A retrospective review of a 10-year single- center experience. Medicine.

[31] Bianco B, Nunes Lipay MV, Guedes AD, Verreschi IT (2008). Clinical implications of the detection of Y-chromosome mosaicism in Turner’s syndrome: report of 3 cases. Fertil Steril.

[32] Bianco B, Lipay M, Guedes A, Oliveira K, Verreschi IT (2009). SRY gene increases the risk of developing gonadoblastoma and/or nontumoral gonadal lesions in Turner syndrome. Int J Gynecol Pathol.

[33] Bianco B, Oliveira KC, Guedes AD, Barbosa CP, Lipay MV, Verreschi IT (2010). OCT4 gonadal gene expression related to the presence of Y-chromosome sequences in Turner syndrome. Fertil Steril.

[34] De Marqui AB, da Silva-Grecco RL, Balarin MA (2016). [Prevalence of Y-chromosome sequences and gonadoblastoma in Turner syndrome]. Rev Paul Pediatr.

[35] Talerman A, Roth LM (2007). Recent advances in the pathology and classification of gonadal neoplasms composed of germ cells and sex cord derivates. Int J Gynecol Pathol.

[36] Asadourian LA, Taylor HB (1969). Dysgerminoma. An analysis of 105 cases. Obstet Gynecol.

[37] Capito C, Arnaud A, Hameury F, Fremond B, Lardy H, Leclair MD (2011). Dysgerminoma and gonadal dysgenesis: the need for a new diagnosis tree for suspected ovarian tumours. J Pediatr Urol.

[38] Subbiah V, Huff V, Wolff JE, Ketonen L, Lang FF, Stewart J (2009). Bilateral gonadoblastoma with dysgerminoma and pilocytic astrocytoma with WT1 GT-IVS9 mutation: A 46 XY phenotypic female with Frasier syndrome. Pediatr Blood Cancer.

[39] Pendergrass EP, Selman J (1946). Dysgerminoma of the ovary with widespread metastases. Radiology.

[40] Brody S (1961). Clinical aspects of dysgerminoma of the ovary. Acta radiol.

[41] Finklestein JZ, Higgins GR, Faust J, Karon M (1972). Serum fetoprotein and malignancy in children. Cancer.

[42] Harilal KR, George KP, Pillai NN (1972). Endodermal sinus tumour (yolksac tumour)of the ovary. J Obstet Gynec India.

[43] Teilum G (1959). Endodermal sinus tumors of the ovary and testis. Comparative morphogenesis of the so-called mesonephroma ovarii (Schiller) and extraembryonic (yolk sac-allantoic) stuctures of the rat’s placenta. Cancer.

[44] Ito K, Kawamata Y, Osada H, Ijichi M, Takano H, Sekiya S (1998). Pure yolk sac tumor of the ovary with mosaic 45X/46X + mar Turner’s syndrome with a Y-chromosomal fragment. Arch Gynecol Obstet.

[45] Madiwale CV, Fernandes GC, Pandit AA, Kane SV (2003). Gonadoblastoma with distinctly unusual pattern of yolk sac tumour overgrowth. J Postgrad Med.

[46] Nam JH, Chung DG, Kim YM, Kim YT, Mok JE (1994). Endodermal sinus tumor arising from a dysgenetic gonad in a 46,XX female combined with mullerian agenesis. Gynecol Oncol.

[47] Riopel MA, Spellerberg A, Griffin CA, Perlman EJ (1998). Genetic analysis of ovarian germ cell tumors by comparative genomic hybridization. Cancer Res.

[48] Looijenga LH, Hersmus R, de Leeuw BH, Stoop H, Cools M, Oosterhuis JW (2010). Gonadal tumours and DSD. Best Pract Res Clin Endocrinol Metab.

[49] Ulbright TM (2005). Germ cell tumors of the gonads: a selective review emphasizing problems in differential diagnosis, newly appreciated, and controversial issues. Mod Pathol.

[50] Norris HJ, Zirkin HJ, Benson WL (1976). Immature (malignant) teratoma of the ovary: a clinical and pathologic study of 58 cases. Cancer.

[51] Levin HS (2000). Tumors of the testis in intersex syndromes. Urol Clin North Am.

[52] Troche V, Hernandez E (1986). Neoplasia arising in dysgenetic gonads. Obstet Gynecol Surv.

[53] Cools M, Wolffenbuttel KP, Drop SL, Oosterhuis JW, Looijemga LH (2011). Gonadal development and tumor formation at the crossroads of male and female sex determination. Sex Dev.

[54] Cools M, Wolffenbuttel KP, Hersmus R, Mendonca BB, Kaprová J, Drop SLS (2017). Malignant testicular germ cell tumors in postpubertal individuals with androgen insensivity: prevalence, pathology and relevance of single nucleotide polimorphism-based susceptibility profiling. Human Reprod.

[55] Bianco B, Lipay MV, Melaragno MI, Guedes AD, Verreschi IT (2006). Detection of hidden Y mosaicism in Turner’s syndrome: importance in the prevention of gonadoblastoma. J Pediatr Endocrinol Metab.

[56] Freriks K, Timmers HJ, Netea-Maier RT, Beerendonk CC, Otten BJ, van Alfen-van der Velden JA (2013). Buccal cell Fish and blood PCR-Y detect high rates of X chromosomal mosaicism and Y chromosomal derivatives in patients with Turner syndrome. Eur J Med Genet.

[57] Queipo G, Aguirre D, Nieto K, Peãa YR, Palma I, Olvera J (2008). Intracranial germ cell tumors: association with Klinefelter syndrome and sex chromosome aneuploidies. Cytogenet Genome Res.

[58] Gravholt CH, Andersen NH, Conway GS, Dekkers OM, Geffner ME, Klein KO (2017). Clinical practice guidelines for the care of girls and women with Turner syndrome: proceedings from the 2016 Cincinnati International Turner Syndrome Meeting. Eur J Endocrinol.

[59] Sallai A, Sólyom J, Dobos M, Szabó J, Halász Z, Ságodi L (2010). Y-chromosome markers in Turner syndrome: Screening of 130 patients. J Endocrinol Invest.

[60] Gravholt CH, Fedder J, Naeraa RW, Muller J (2000). Occurrence of gonadoblastoma in females with Turner syndrome and Y chromosome material: a population study. J Clin Endocrinol Metab.

[61] Klaassen Z, Reinstatler L, Wilson SN, Elington C, Li Q, Terris MK (2016). Clinical Disparities for Minorities and Foreign-Born Men With Undescended Testicular Germ Cells Tumors. Clin Genitourin Cancer.

[62] O’Shaughnessy PJ (2014). Hormonal control of germ cell development and spermatogenesis. Semin Cell Dev Biol.

[63] Cheng L, Sung MT, Cossu-Rocca P, Jones TD, MacLennan GT, De Jong J (2007). OCT4: biological functions and clinical applications as a marker of germ cell neoplasia. J Pathol.

[64] Palma I, Garibay N, Pena-Yolanda R, Contreras A, Raya A, Dominguez C (2013). Utility of OCT3/4, TSPY and β-catenin as biological markers for gonadoblastoma formation and malignant germ cell tumor development in dysgenetic gonads. Dis Markers.

[65] Luo X, Ikeda Y, Parker KL (1994). A cell-specific nuclear receptor is essential for adrenal and gonadal development and sexual differentiation. Cell.

[66] Biason-Lauber A (2012). WNT4, RSPO1, and FOXL2 in sex development. Semin Reprod Med.

[67] de Jong J, Stoop H, Gillis AJ, van Gurp RJ, van de Geijn GJ, Boer Md (2008). Differential expression of SOX17 and SOX2 in germ cells and stem cells has biological and clinical implications. J Pathol.

[68] Rapley EA, Turnbull C, Al Olama AA, Dermitzakis ET, Linger R, Huddart RA (2009). A genome-wide association study of testicular germ cell tumor. Nat Genet.

[69] Mirabello L, Kratz CP, Savage AS, Greene MH (2012). Promoter methylation of candidate genes associated with familial testicular cancer. Int J Mol Epidemiol Genet.

[70] Spoor JA, Oosterhuis JW, Hermus R, Biermann K, Wolffenbuttel KP, Cools M (2018). Histological Assessment of Gonads in DSD: Relevance for Clinical Management. Sex Dev.

[71] Lau YF (1999). Gonadoblastoma, testicular and prostate cancers, and the TPSY gene. Amer J Hum Genet.

[72] Hersmus R, Van Bever Y, Wolffenbuttel KP, Biermann K, Cools M, Looijenga LH (2017). The biology of germ cell tumors in disorders of sex development. Clin Genet.

[73] Looijenga LH, Stoop H, der Leeuw HP, de Gouveia Brazao CA, Gillis AJ, van Roozendaal KE (2003). POU5F1 (OCT3/4) identifies cells with pluripotent potential in human germ cell tumors. Cancer Res.

